# Action of swim-up and caffeine on equine frozen sperm

**DOI:** 10.1590/1984-3143-AR2022-0056

**Published:** 2022-11-21

**Authors:** Natália de Castro Alves, Soraia de Araújo Diniz, Rodrigo Novaes Viegas, Ana Luiza Arigoni, Marina Morra Freitas, Ângela Quintão Lana, Monique de Albuquerque Lagares

**Affiliations:** 1 Escola de Veterinária, Universidade Federal de Minas Gerais, Belo Horizonte, MG, Brasil

**Keywords:** spermatozoa, cryopreservation, freezing, sperm selection, stallion

## Abstract

Cryopreservation of equine semen is crucial to semen commercialization. However, it reduces sperm motility and longevity. Thus, sperm selection methods and addition of motility-activating substances to sperm, such as caffeine, may improve sperm quality of equine frozen semen. The objective of the current work was to evaluate the effects of caffeine on recovery and quality parameters of frozen-thawed sperm subjected to swim-up selection to be used in intracytoplasmic sperm injection (ICSI) in assisted reproductive techniques. Stallion semen were frozen and after thawing different caffeine concentrations were added to the samples performing four treatments control (no caffeine), 3, 5, and 7.5 mM caffeine. Sperm kinematic and motility were assessed by computer-assisted sperm analysis (CASA). Then, the four treated samples were submitted to the swim-up sperm selection, and the number of recovered sperm and morphology were evaluated at four times 20, 40, 60, and 80 min. The swim-up increased the recovery proportion of normal morphology sperm without (80.1±1%) or with caffeine addition (3mM: 81.2±1%, 5mM: 79.9±1% and 7.5 mM 78.9±1%) compared to the thawed semen (70±2%). However, the addition of 5 mM caffeine induced an increase in sperm motility (38.9±2.8 vs. 32.6±3.4%, P<0.05), and sperm recovery after swim-up (7.9x10^6^ vs. 3.4x10^6^ sperm/ml, P<0.05) compared to the control. The addition of 5 mM caffeine to frozen-thawed equine semen before swim-up selection improved sperm motility and increased the sperm recovery rate while not decreasing the percentage of morphologically normal sperm. Thus, caffeine addition to frozen-thawed equine semen before swim-up selection has potential clinical application in improving sperm quality for use in ICSI.

## Introduction

Cryopreserved equine semen can be stored almost indefinitely, facilitating its commercialization, irrespective of the location of the stallion or mare. It also allows semen preservation of sires with superior genetic merit (Brandão et al., 2006). However, fertility following artificial insemination of cryopreserved equine semen is highly variable (Voss, 1993; Squire et al., 1999). During semen cryopreservation, temperature changes and oxidative stress damage sperm (Tash and Mann, 1973), compromising cell viability, motility, and longevity (Roca et al., 2013).

Sperm selection have been used to select the best quality sperm from poor quality semen samples after thawing (Podico et al., 2020). The choice of sperm cell selection techniques depends on sperm concentration and the recovery of highly functional sperm cell population. There are various methods of sperm selection, including dilution and washing (centrifugation and resuspension), sperm migration (swim-up), selective washing of subpopulations including density gradient centrifugation, e.g., Percoll (Morrell, 2012), and addition of adhesive substances to eliminate dead sperm and debris (Larentis and Bastos, 2019). Ideally, sperm selection should: isolate as many motile sperm as possible, not alter or damage sperm, remove dead sperm, and enable processing of large volumes (Larentis and Bastos, 2019).

A selection method as swim-up improves quality of stallion semen by selecting sperm with progressive motility and sperm normal morphology (Hoogewijs et al., 2012). Furthermore, since this method selects sperm with fewer defects, it can increase sperm longevity (Mbizvo et al., 1993). When applied to stallion semen, swim-up allows the selection of a population of sperm which exhibit sperm motility, viability, normal morphology, mitochondrial membrane potential, and membrane integrity (Colleoni et al., 2011). However, the number of recovered sperm after swim-up are still low. Therefore, alternatives to increase the number of recovered sperm after swim-up are of interest.

Strategies to maximize the success of artificial insemination (AI) with frozen-thawed stallion sperm are of great importance to the industry (Maia and Bicudo, 2009). A combination of sperm motility enhancers and sperm swim-up selection method should be considered to increase recovery of motile and morphologically normal frozen-thawed sperm for stallions, particularly those with poor semen quality.

**Figure 1:**
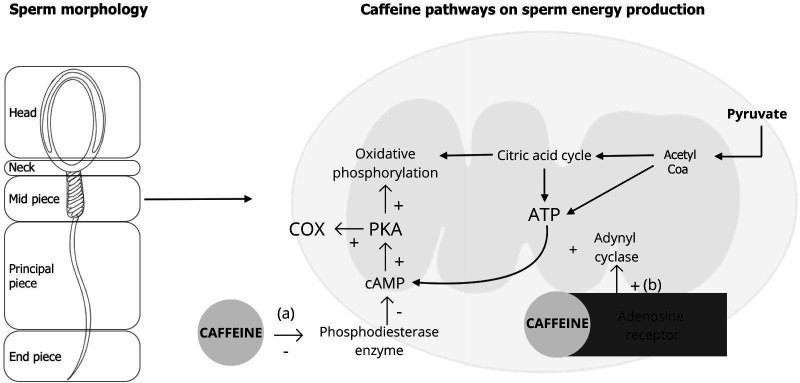
Scheme of the pathways of caffeine action on sperm energy production inhibiting phosphodiesterase enzyme activity on cAMP degradation (a) and binding to the adenosine receptor stimulating adenylyl cyclase converting ATP into cAMP (b). Both pathways cause an increase of intracellular concentration of cAMP activating protein kinase (PKA), increasing cytochrome c oxidase (COX) activity and oxidative phosphorylation.

Adding caffeine to semen increases sperm motility and longevity (Maia and Bicudo, 2009). Caffeine, 1,3,7-trimethylxanthine, is a bioactive substance with antioxidant properties (Pariz and Hallak, 2016). It activates sperm motility by inhibition of phosphodiesterase, which converts cAMP into its acyclic form, which in turn activates protein kinase (Huo et al., 2002) (Figure 1). In this manner, caffeine increases cAMP half-life and in sperm stimulates motility, cyclic hyperactivation, capacitation, and the acrosome reaction (Mbizvo et al., 1993). Early work in livestock has demonstrated that the addition of 2.5 mM caffeine increased motility of bovine sperm selected by swim-up (Correa and Zavos, 1996). Increased sperm motility and fertility associated with a decreased sperm nitrite concentration was reported when 5 mM caffeine concentration was added to frozen-thawed stallion (Alves et al., 2021).

The efficiency of frozen semen is greatly increased by ICSI, as only a few sperm are needed, from which one is selected to fertilize each oocyte. If motility and recovery of normal morphologically sperm is good, a portion of this straw can be used at a time, for fertilization of numerous oocytes, thus allowing an existing store of frozen semen to produce many embryos (Lazzari et al., 2002). Application of ICSI to equine oocytes provided that a motile sperm is selected for injection (Lazzari et al., 2002). However, when sperm from stallions of low fertility were used, lower cleavage rates and lower development was achieved compared to stallions of proven fertility (Colleoni et al., 2009). According to these results a more efficient selection technique for the most fertile sperm would be an interesting option to improve the efficiency of embryo production. This is especially critical in the case of ICSI whereby all selective barriers for sperm are bypassed. Those later barriers are presented in IVF such as cumulus penetration, membrane recognition and membrane fusion (Pérez-Cerezales et al., 2018).

Caffeine addition to equine sperm before swim-up is an attractive alternative to select sperm with high metabolic rates for *in vitro* production of equine embryos by intracytoplasmic sperm injection [ICSI, Galli et al. (2003)] since it uses selected high-quality stallion sperm of frozen-thawed semen.

The objective of the present study was to significantly increase both the *in vitro* sperm recovery rate and percentage of morphologically normal sperm of cryopreserved equine semen treated with caffeine and subjected to swim-up sperm selection after thawing for use in assisted reproductive techniques (ART).

## Methods

All experimental procedures were performed according to Brazilian ethical and animal welfare principles for the utilization and care of animals used in research and were approved by the ethical committee (Comissão de Ética no Uso de Animais, CEUA) at the Federal University of Minas Gerais (UFMG), protocol 394/2017.

### Semen collection and evaluation

Semen from nine stallions (one ejaculate from each stallion) was collected with an artificial vagina in March, October, November, and December. The stallions were Mangalarga Marchador, Arabian, and Campolina breeds, 5 to 6 years old, from stud farms near Belo Horizonte in Minas Gerais, Brazil. Sperm progressive motility (PM) was evaluated by bright-field microscopy (x100) and only ejaculates with PM ≥ 50% and vigor ≥ 3 were used. Sperm concentrations were measured with a hemocytometer. Sperm morphology was assessed with phase-contrast microscopy (x1,000) after the semen was put in a buffered formaldehyde saline [wet mount preparation, Mies (1975)]. Two hundred sperm were evaluated per sample and only ejaculates with ≥70% morphologically normal sperm were used (CBRA, 2013).

### Semen freezing

Semen was initially diluted (1:1) with Kenney extender (Kenney et al., 1975) and centrifuged (450 × g, 10 min). For freezing, sperm were resuspended in INRA82 extender with 2% egg yolk and 2.5% glycerol (Pillet et al., 2008) to a final concentration of 100 × 10^6^ sperm/mL, packaged in 0.5 mL straws, and cooled to 5 °C (0.27 °C/min). For semen freezing, straws were placed 2.5 cm above liquid nitrogen for 20 min and then plunged into it.

### Sperm motility and kinematic analysis

Straws were thawed at 37 °C for 30 s and divided into four treatments: 0 (Control), 3, 5, and 7.5 mM caffeine (Sigma- Aldrich 27602). Sperm motility was assessed with computer-assisted sperm analysis (CASA, Sperm Class Analyzer, SCA® 2005 VS 4.0.0 Microptik S.L., Barcelona, Spain). One straw from each treatment was thawed at 37 °C for 30 s and the sperm analyzed for the following motility characteristics: velocity curvilinear (VCL μm/s), velocity straight line (VSL μm/s), velocity average path (VAP μm/s), linearity (LIN %), straightness (STR %), wobble (WOB %), amplitude of lateral head displacement (ALH μm), beat-cross frequency (BCF Hz), and percentage total motility (TM). A 5-μl semen sample was immediately placed on a slide, covered with a coverslip (22 × 22 mm) and observed with a phase contrast microscope at 100× magnification with a warm plate at 37°C linked to the CASA. A total of nine fields per sample were analyzed. The CASA set-up was capture: 25 images per second; optics: Ph-; particle area greater than 4 and smaller than 75 μm^2^; curvilinear velocity slow: smaller than 10, medium: between 45 and 90, and rapid: greater than 90 μm/s; progressive motility: greater than 75%, and straightness and circular motility: smaller than 50% linearity. The samples were evaluated by CASA immediately and after 20, 30, 40, and 50 min after thawing and caffeine addition.

### Sperm selection, recovery, and morphology analysis

**Figure 2:**
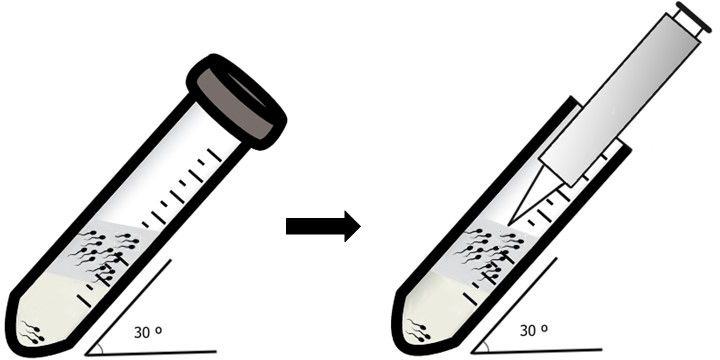
Scheme of swim-up.

Motile sperm were selected by swim-up. For this, 0.3 mL semen was placed in a 1.5 mL Eppendorf conical tube containing 0.9 mL Tissue culture medium 199 (TCM 199) with Hank's buffered salt solution and 10% FBS. To perform the swim-up, tubes were placed at 30 °C for 20, 40, 60, and 80 min in a water bath at 37 °C (Figure 2). After incubation, 0.4 mL supernatant were removed with a pipette (~1/3 of the total volume) and 10 µL was preserved in 100 µL 2% buffered formaldehyde saline (1:10 dilution) to calculate sperm concentration. The remaining supernatant (~0.4 mL) was preserved in 2% buffered formaldehyde saline to evaluate sperm morphology. Sperm concentration was determined with a hemocytometer under bright field microscopy at 400× magnification and morphologies of 200 sperm per sample were evaluated with phase-contrast microscopy at 1000× magnification.

### Statistical analyses

The experiment was performed in a randomized block design, defining stallion as the block factor. Variance analysis (ANOVA) was performed for sperm motility and kinematic parameters, sperm recovery and morphologically normal sperm after the swim-up. For all analyses, P<0.05 was considered significant and Duncan’s test was used to locate differences. When there was no significant interaction between treatments and time, marginal means were compared and presented in tables. Otherwise, conditional means were compared. All data were analyzed using the Infostat program (FCA, Universidad Nacional de Córdoba, Argentina).

## Results

As the evaluated CASA parameters showed no interaction between treatment and time (p > .05), only the treatment mean values are presented in Table 1. Adding 5 mM caffeine increased TM compared to the control (38.9 ± 2.8 vs 32.6 ± 3.4%, P< 0.05, Table 1). The VCL of the 7.5 mM caffeine sample was significantly higher than that of 3 mM caffeine, although it did not differ from the control or 5 mM caffeine (P> 0.05). Addition of 7.5 mM caffeine increased BCF values compared to control (8.6 ± 0.3 vs 7.4 ± 0.3, P<0.05).

**Table 1:** CASA end points (mean ± SEM) after adding caffeine to frozen-thawed stallion semen.

**Treatment (mM caffeine)**	**TM (%)**	**VAP (µm/s)**	**VSL (µm/s)**	**VCL (µm/s)**	**BCF (Hz)**	**STR (%)**	**LIN (%)**	**WOB (%)**	**ALH (µm)**
0	32.6 ± 3.4^b^	23.0 ± 1.3^a^	17.2 ± 1.1^a^	31.4± 1.5^ab^	7.4 ± 0.3^b^	72.6 ± 1.3^a^	52.8 ± 1.3^a^	71.8 ± 1.4^a^	2.1 ± 0.1^a^
3	27.2 ± 1.9^b^	21.2 ± 0.9^a^	15.9 ± 0.8^a^	29.6± 1.0^b^	7.6 ± 0.3^b^	73.3± 1.1^a^	52.2 ± 1.4^a^	70.7 ± 1.1^a^	2.1 ± 0.1^a^
5	38.9 ± 2.8^a^	24.3 ± 1.1^a^	18.4 ± 0.9^a^	33.5± 1.3^ab^	8.0 ± 0.3^ab^	74.3± 1.1^a^	53.7 ± 1.5^a^	71.7 ± 1.1^a^	2.2 ± 0.1^a^
7.5	33.6 ± 2.8^ab^	24.9 ± 1.6^a^	18.5 ± 1.3^a^	35.3± 1.9^a^	8.6 ± 0.3^a^	72.9 ± 1.3^a^	50.8 ± 1.7^a^	68.9 ± 1.4^a^	2.1 ± 0.1^a^

ab: Within a column, values without a common superscript differed (P <0.05).

MT: total motility, VAP: average path velocities, VSL: straight line, VCL: curvilinear speed, BCF: beat cross frequency, STR: straightness, LIN: linearity, WOB: wobble, ALH: amplitude of lateral head displacement.

Duration of swim-up did not influence the number of sperm recovery (P>0.05, Table 2). The mean number of sperm after thawing was 100 ± 1.1 × 10^6^ sperm/mL (mean ± SD). After swim-up, sperm recovery was 3.4% (3.4 ± 0.7 × 10^6^ sperm/mL); this was more than doubled with addition of 5 mM caffeine (7.9±1.7x10^6^/mL, P<0.05; Table 2).

**Table 2:** Concentrations (mean ± SD) of frozen-thawed stallion sperm recovered after varying intervals of swim-up and addition of various concentration of caffeine.

**Caffeine (mM)**	**Recovered sperm (× 10^6^ sperm/mL)**				
	**20 min**	**40 min**	**60 min**	**80 min**	**Mean**
0	3.5±1.1	4.9±2.0	3.5±1.2	1.7±0.4	3.4±0.7^b^
3	3.4±0.8	3.7±1.2	10.1±4.7	5.7±2.4	5.7±1.4^ab^
5	8.2±3.7	9.7±4.2	5.9±2.7	7.7±4.1	7.9± 1.7^a^
7.5	3.5±0.9	5.4±2.2	6.7±3.1	5.8±2.3	5.3± 1.1^ab^
Mean	4.6±1.6	5.9±2.4	6.5±2.9	5.2±2.3	

ab: Within a column, values without a common superscript differed (P <0.05).

Post thaw, 70.0 ± 2.1% of sperm were morphologically normal, with an ~10 percentage point increases due to swim-up with no caffeine added (80.1 ± 1.0%), or swim-up plus caffeine (means, 78.9 to 81.2%, Table 3). Swim-up reduced the proportion of bent-tail sperm compared to thawed semen (P < 0.05). However, the proportion of sperm with midpiece defects, head defects or proximal and or cytoplasmic droplets after swim-up did not decrease in comparison to thawed semen (P > 0.05).

**Table 3:** Percentage (mean ±SD) of morphologically normal or abnormal frozen-thawed stallion sperm prior to swim-up and post swim-up with addition of different concentration of caffeine.

**Sperm morphology (%)**						
**Treatment**	**Normal**	**Midpiece**	**Bent tail**	**Head**	**Proximal CD**	**Distal CD**
Thawed semen	70.0 ± 2.1^b^	7.2 ± 3.8^ab^	9.0 ± 2.4^a^	5.4 ± 4.3^a^	4.4 ± 3.7^a^	4.0 ± 4.2^a^
0 mM	80.1 ±1.0^a^	6.5 ±2.1^b^	1.1 ± 1.1^b^	5.5 ± 2.7^a^	4.0 ±2.9^a^	2.8 ± 2.1^a^
3 mM	81.2 ±1.0^a^	8.1 ±3.5^ab^	1.0 ± 1.9^b^	5.1 ± 2.2 ^a^	2.7 ±2.3^a^	1.9 ± 1.7^a^
5 mM	79.9 ± 1.0^a^	10.4 ± 3.6^a^	1.2 ± 1.8^b^	3.8 ± 2.8 ^a^	2.9 ± 2.6^a^	1.8 ± 2.1^a^
7.5 mM	78.9 ± 1.0^a^	9.3 ± 4.0^ab^	0.6 ± 1.1^b^	4.5 ± 2.9 ^a^	3.9 ± 2.4^a^	2.8 ± 2.6 ^a^

ab: Within a column, means without a common superscript differed (P < 0.05).

Midpiece = Midpiece defects, Head = Head defects, Proximal CD = Proximal cytoplasmic droplets, and Distal CD = Distal cytoplasmic droplets.

## Discussion

In the present study, 5 mM caffeine increased sperm motility and more than doubled sperm recovered compared to that in the control, without caffeine. Adding 2 mM caffeine increased motility of cooled equine semen (Carrington et al., 2011) but was not beneficial for frozen-thawed semen (Stephens et al., 2013). Conversely, adding 5 mM caffeine to frozen-thawed stallion semen increased sperm motility and fertility, associated with an antioxidant function (Alves et al., 2021).

Density gradient centrifugation and swim-up are among the most used sperm selection techniques in clinical practice (Rappa et al., 2016). Both methodologies are intended to isolate viable sperm. Density gradient centrifugation tends to concentrate motile sperm in bottom layers while seminal plasma, debris, round cells, dead sperm, and immature sperm are retained in the upper layers (Henkel, 2012) Sperm with compact chromatin and at least reasonable motility can reach the bottom of the conical tube even in the presence of mitochondrial impairment and DNA damage. Therefore, density gradient centrifugation can successfully retain only immature forms, concentrating mature and morphologically normal forms, which might increase the risk of choosing a DNA-fragmented sperm at the time of ICSI (Muratori et al., 2019)

The use of swim-up with human sperm provide a sorted sperm subpopulation with increased viability, motility, morphology, DNA integrity and reduced percentage of apoptotic sperm (Kim et al., 2015). In stallions, swim-up improved sperm motility and with normal morphology in raw semen (Morrell et al., 2009).

Sperm selection can be used for *in vitro* production of equine embryos by ICSI (Landim-Alvarenga et al., 2008). Among conventional techniques for sperm preparation in ART procedures, the swim-up technique, is currently considered a well-established and efficient method. The swim-up technique was analyzed and was found to be the technique that causes the lowest DNA fragmentation rate in human sperm (Volpes et al., 2016) and is suggested to be the best option in terms of low cost and reduced time. In stallions, swim-up was associated with higher cleavage and blastocyst rates after ICSI when compared to single layer density gradient centrifugation alone (Choi et al., 2016).

In the present study, all swim-up treatments, including 5 mM caffeine, decreased the proportion of bent tails. Conversely, no decrease of sperm with head defects or proximal and or cytoplasmic droplets was observed, whereas 5 mM caffeine increased the proportion of sperm with midpiece defects after swim-up compared to the control.

In sperm porcine similar results regarding effect of swim-up on decreasing the bent tails was reported (Navarro-Serna et al., 2021). Swim-up selects a high proportion of sperm with normal morphology (Rodríguez-Martinez et al., 1997), based on migration of progressively motile sperm (Hoogewijs et al., 20012). During semen cryopreservation, there can be increased sperm with bent tails due to cold shock, thereby reducing post-thaw sperm quality (Watson, 2000). In this study, the decreased proportion of bent tails after swim-up may have led to an increase of the proportion of morphologically normal sperm, since no other sperm defects were decreased. Furthermore, 5 mM caffeine increased sperm midpiece defect, possibly because of the sperm motility increase.

In this study, semen samples were frozen with 100 x 10^6^ sperm/ mL in 0.5 mL straws, so that theoretically two semen straws could be subjected to swim-up with 5 mM caffeine to obtain 7.9 x 10^6^ sperm. Besides swim-up is a simple and cost-effective sperm selection it is particularly useful to select sperm from poor-quality frozen-thawed semen samples (García et al., 2009).

## Conclusion

In conclusion, adding 5 mM caffeine to equine sperm before swim-up was an attractive alternative to increase recovered sperm number and select sperm with high motility, thereby enabling its use for ICSI. Further, sperm selected by swim-up associated with caffeine has promising implications as a selection method for basic sperm studies and ARTs both in human and veterinary clinical practice.
